# Complex long-distance effects of mutations that confer linezolid resistance in the large ribosomal subunit

**DOI:** 10.1093/nar/gkv729

**Published:** 2015-07-21

**Authors:** Simone Fulle, Jagmohan S. Saini, Nadine Homeyer, Holger Gohlke

**Affiliations:** Institute for Pharmaceutical and Medicinal Chemistry, Department of Mathematics and Natural Sciences, Heinrich-Heine University, Universitätsstrasse 1, 40225 Düsseldorf, Germany

## Abstract

The emergence of multidrug-resistant pathogens will make current antibiotics ineffective. For linezolid, a member of the novel oxazolidinone class of antibiotics, 10 nucleotide mutations in the ribosome have been described conferring resistance. Hypotheses for how these mutations affect antibiotics binding have been derived based on comparative crystallographic studies. However, a detailed description at the atomistic level of how remote mutations exert long-distance effects has remained elusive. Here, we show that the G2032A-C2499A double mutation, located > 10 Å away from the antibiotic, confers linezolid resistance by a complex set of effects that percolate to the binding site. By molecular dynamics simulations and free energy calculations, we identify U2504 and C2452 as spearheads among binding site nucleotides that exert the most immediate effect on linezolid binding. Structural reorganizations within the ribosomal subunit due to the mutations are likely associated with mutually compensating changes in the effective energy. Furthermore, we suggest two main routes of information transfer from the mutation sites to U2504 and C2452. Between these, we observe cross-talk, which suggests that synergistic effects observed for the two mutations arise in an indirect manner. These results should be relevant for the development of oxazolidinone derivatives that are active against linezolid-resistant strains.

## INTRODUCTION

**Figure 1.**
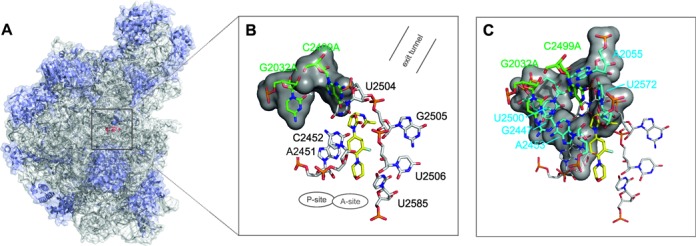
Binding region of linezolid in H50S. (**A**) Structure of the large ribosomal subunit (PDB code 3CPW (8)). The ribosomal RNA is shown in gray and the protein chains are shown in blue; the binding position of linezolid (red) is depicted by a black square. (**B** and **C**) Binding mode of linezolid in the PTC of H50S. Nucleotides forming the first (black labels) and second shell (light blue labels) of the binding site are depicted in B and C, respectively; the two mutation sites (G2032A and C2499A) are highlighted in green. The locations of the A- and P-site and of the exit tunnel are indicated.

The ever increasing emergence of multidrug-resistant bacteria will make current antibiotics virtually ineffective in the future. This stresses the need to identify novel classes of antibiotics (1,2). Yet, only compounds of five new classes of antibiotics have been approved by the FDA in the past 30 years, among them antibiotics of the oxazolidinone class (3). Oxazolidinone antibiotics display bacteriostatic activity against many important pathogens, including methicillin-resistant *Staphylococcus aureus* (MRSA) and vancomycin-resistant *Enterococcus faecium* (VREF) (4). So far, only linezolid has been approved for therapeutic use (5) but enhanced oxazolidinones are currently undergoing clinical evaluation (3,6). The co-crystal structures of linezolid with the large ribosomal subunits of the eubacterium *Deinococcus radiodurans* (D50S) (7) and the archaeon *Haloarcula marismortui* (H50S) (8) (Figure 1A) demonstrate that the antibiotic exerts its action by binding to the A-site of the highly conserved peptidyl transferase center (PTC) (7,8) (Figure 1B) and preventing the proper placement of the incoming aminoacyl-tRNA. As a consequence, protein synthesis is inhibited.

Not long after the commercial release of linezolid, strains of MRSA and VREF appeared in the clinics that are resistant against linezolid (6,8). Also considering nucleotides conferring linezolid resistance in other bacterial strains (see Supplementary Table S1 and (7,9)), it was revealed that many of these are clustered in a distinct region of the PTC (called the PTC ‘rear wall’) and are located in a distance of 6–12 Å from the affected antibiotic (9,10). An explanation for this observation is that mutations of the highly conserved linezolid binding pocket are likely unfavorable for ribosome function, (7,10) while nucleotide alterations in more remote regions of lower sequence conservation bear a lower potential to affect ribosome function lethally.

Oxazolidinones bind to and inhibit both bacterial and archaeal ribosomes (7,8) but do not interact with human cytoplasmic ribosomes (7). Notably, out of 10 mutations known to give rise to linezolid resistance in bacteria and archaea, the nucleotides corresponding to two of these mutations are already present in the 28S rRNA of *Homo sapiens* at positions 2032 and 2499 (*Escherichia coli* (*E. coli*) numbering used throughout this manuscript) (7,9). Mutations at these positions also mediate resistance against other antibiotics (9–12). Both mutation sites, which are either highly or absolutely conserved in eubacteria (position G2032: 94%; C2499: 100%), (9) are more than 10 Å away from linezolid bound at the PTC and constitute third shell nucleotides with respect to the linezolid binding pocket. Thus, mutation effects must propagate to nucleotides forming direct interactions with the drug (first shell nucleotides) via second shell nucleotides (Figure 1B,C) (9). Experimental results indicate that single mutations at that distance are not sufficient to confer resistance (9) and that the development of antibiotics resistance due to remote nucleotides requires the additive or even synergistic effect of several mutations (12). Accordingly, a double mutation at these sequence positions (G2032A-C2499A) observed in *Mycobacterium smegmatis* showed remarkable synergistic effects on linezolid resistance relative to the effects of the corresponding single mutations (10,13). Overall, this makes these sites ideal prototypes for investigating how mutations can confer long-distance effects on antibiotics binding.

To gain insights at an atomistic level into how remote mutations exert long-distance effects that lead to resistance to oxazolidinones, we extended a previous study on the determinants of the species selectivity of oxazolidinone antibiotics, which had considered the wild-type structure of H50S (linezolid-H50S_wt_), (14) by performing molecular dynamics (MD) simulations in combination with molecular mechanics Poisson-Boltzmann surface area (MM-PBSA) free energy calculations of linezolid bound to the double mutant G2032A-C2499A of H50S (linezolid-H50S_mut_). Furthermore, MD simulations of two novel oxazolidinone antibiotics, radezolid and tedizolid, that show activity against linezolid-resistant strains, (15) are performed. To the best of our knowledge, this is the first study that investigates resistance to oxazolidinone binding to the 50S ribosomal subunit by simultaneously considering structural, energetic and dynamic aspects. These determinants are consistent in describing effects of a complex but balanced reorganization in the network of inter-nucleotide interactions that percolates from the mutation sites to the PTC. Cross-talk identified between the two main routes of information transfer can explain the experimentally observed synergy of the double mutation. These findings go beyond current knowledge on the structural basis for oxazolidinone resistance. Since antibiotics binding to the PTC share overlapping binding sites, we will finally discuss to what extent insights obtained in this study also relate to other incidences of (cross)-resistance.

## MATERIALS AND METHODS

### Preparation of starting structures

Starting structures for the MD simulations were generated based on the X-ray structure of linezolid in complex with the large ribosomal subunit of *H. marismortui* (H50S) (PDB code 3CPW) solved at a resolution of 2.7 Å (7,8). Details of the setup and simulation of the wild-type structure (linezolid-H50S_wt_) have been described before (14). To investigate the influence of the G2032A-C2499A double mutation on linezolid binding, a model structure (linezolid-H50S_mut_) was generated from the linezolid-H50S_wt_ crystal complex structure by mutating G2032 and C2499 to adenine, respectively. In both structures the CCA-*N-*acetylphenylalanine (CCA-Phe), an analogue of the portion of aminoacyl and peptidyl tRNAs located at the P-site, was removed because the orientation of linezolid in H50S is unaffected by the presence of CCA-Phe (8). For comparison, MD simulations of two novel oxazolidinones (i.e. radezolid (6) and tedizolid (16)) bound to H50S with the double mutation G2032A-C2499A were performed. The starting structures radezolid-H50S_mut_ and tedizolid-H50S_mut_ were generated by changing linezolid in linezolid-H50S_mut_ to the respective oxazolidinone.

### Setup of MD simulations

MD simulations were performed with the Amber 10 and Amber 14 suite of programs (17) using the ff99SB modifications (18) of the Cornell *et al*. force field (19) for the ribosomal structure and the general Amber force field (GAFF) (20) for linezolid, radezolid and tedizolid. Comparison of linezolid structures optimized either with these parameters at the molecular mechanics level or at the Hartree-Fock level yielded very small root mean-square deviations for bond lengths and angles, respectively; furthermore, in general good correlations were found between the quantum mechanical and molecular mechanical energy profiles for rotations around critical torsion angles (Supplementary Figures S1 and S2). See Supplementary Data for details. Atomic charges for linezolid, radezolid and tedizolid were obtained by the RESP (21) procedure using Gaussian 03 (22) and Gaussian 09 (23) and the Antechamber suite (24). All Mg^2+^ ions resolved in the X-ray structure (8) were retained, because they are essential for maintaining the stability of the structures of ribosomes (25). Non-bonded parameters for Mg^2+^ were taken from Åqvist (26). To neutralize the complexes, Na^+^ counter ions were added. Then, the systems were solvated in a truncated octahedral of TIP3P water molecules, (27) forming a solvent shell of at least 10 Å around the solute. The systems were minimized by 250 steps of steepest descent minimization followed by 250 steps of conjugate gradient minimization. After minimization the systems were heated from 100 K to 300 K using canonical ensemble (NVT) MD. The solvent density was then adjusted using isothermal-isobaric ensemble (NPT) MD. Harmonic restraints with force constants of 5 kcal mol^−1^ Å^−2^ were applied to all receptor and ligand atoms in all minimization and equilibration runs. During equilibration it proved necessary to re-solvate the tunnel region multiple times, as water molecules continued to fill voids initially present in the ribosomal structure. Finally, the force constants of the harmonic restraints on the receptor and ligand atoms were gradually reduced from 5 kcal mol^−1^ Å^−2^ to zero over 250 ps in the NVT ensemble. To relax the system without restraints, an additional unrestrained NVT MD was performed for 50 ps at 300 K using a time constant of 2.0 ps for heat bath coupling. In addition, 10 independent replicates of the linezolid-H50S_wt_ and linezolid-H50S_mut_ MD simulations, respectively, were performed for control. For this, the final snapshot of the respective equilibration step was simulated for 20 ps at a slightly increased temperature (i.e. 300.1 K, 300.2 K, …, 301.0 K). The initial MD simulations of linezolid-H50S_wt_ and linezolid-H50S_mut_ were used for the main structural and energetic analyses.

The production runs of all simulations were run at 300 K and achieved lengths of 50 ns of which snapshots saved at 20 ps intervals during the last 20 ns and 10 ns were used for structural and energetic analysis, respectively. In all MD simulations periodic boundary conditions were applied using the particle mesh Ewald method to treat long-range interactions (28). Bond lengths involving bonds to hydrogen atoms were constrained by SHAKE (29). A time step of 2 fs was used for the integration of the equations of motion, and a direct-space non-bonded cutoff of 9 Å was applied.

The MD simulations were performed on the supercomputer JUROPA at the Jülich Supercomputing Center and on an in-house compute cluster with GPGPUs.

### Structural analysis of MD trajectories

The ‘ptraj’ module of Amber 10 (17) was used for analyzing the root-mean square deviation (RMSD) between pairs of structures, the root-mean square fluctuations (RMSF) about the mean position of atoms, and the formation of hydrogen bonds and aromatic ring interactions. For investigating structural deviations along the trajectories, the RMSD of all residues of the linezolid-H50S complexes as well as the RMSD of the ‘core’ residues were computed with respect to the starting structure of the respective production run (see (14) for more details). RMSF values were calculated after aligning all residues of the H50S structure that are 10 Å around linezolid in the 3CPW starting structure. Hydrogen bonds were defined by a distance cutoff of 3.2 Å and an angle cutoff of 120° and were only considered if their occupancies attained >60% (percent of simulation time in which the hydrogen bond is formed). Aromatic stacking interactions between two nucleobases were defined by a distance cutoff of 5.0 Å from one ring center to another (8,9) and only considered if their occupancies attained >60%.

### Binding free energy calculations

Computational methods that combine molecular mechanics energy and implicit solvation models, such as the MM-PBSA approach, (30–32) have been widely exploited in free energy calculations. Compared with rigorous methods such as free energy perturbation and thermodynamic integration, (33) the MM-PBSA method is computationally more efficient (34). Therefore the MM-PBSA approach was used in this study to investigate the energetic determinants of binding of linezolid to the wild-type H50S and the G2032A-C2499A mutant. The MM-PBSA method estimates the free energy of a molecule *x* as the sum of its gas-phase energy (*H^x^*_gas_), solvation free energy (*G^x^*_solv_) and entropy (Equation 1).

(1) \begin{equation*} G^{\rm x} (i) = H_{{\rm gas}}^{\rm x} (i) + G_{{\rm solv}}^{\rm x} (i) - TS^{\rm x} (i) \end{equation*}

Contributions due to changes in the solute entropies were not considered here. Therefore, all values reported for the MM-PBSA calculations should be considered as ‘effective energies’ (Δ*G*_effective_) (35). The effective binding energies were computed as the differences of the effective energies of the complex and the receptor and ligand (Equation 2).

(2) \begin{equation*} \Delta G_{{\rm effective}}^{{\rm total}} {=} < G_{{\rm effective}}^{{\rm complex}} (i) {-} G_{{\rm effective}}^{{\rm receptor}} (i) {-} G_{{\rm effective}}^{{\rm ligand}} (i) >\end{equation*}

<·> denotes an average over snapshots *i* from the MD trajectories. In the single-trajectory MM-PBSA approach employed here, the snapshots are extracted from a single simulation of the complex (35,36). In all MM-PBSA calculations, 500 snapshots extracted from the last 10 ns of the production runs of the MD simulations, i.e. snapshots recorded in intervals of 20 ps, were used. Magnesium ions were considered in the calculations because of their role in imparting overall stability to the H50S system. For each snapshot, gas-phase energies *H^x^*_gas_*(i)* were calculated by summing up contributions from internal energies (including bond, angle and torsion angle energies), electrostatic energies and van der Waals energies using the ff99SB modifications (18) of the Cornell *et al*. force field (19) with no cutoff. Solvation free energies *G^x^*_solv_*(i)* were computed as the sum of polar and non-polar contributions. The polar contribution to the solvation free energy was calculated by the Poisson-Boltzmann (PB) model (see below). The non-polar contribution to the solvation free energy was estimated by a solvent-accessible surface area (SASA)-dependent term:

(3) \begin{equation*} G_{{\rm nonpolar}}^x (i) = \gamma {\rm SASA}^x (i) + b \end{equation*}

The SASA*^x^(i)* was determined with the linear combinations of pairwise overlaps (LCPO) method (37) as implemented in Amber 10. For the calculation of the non-polar contribution to the solvation free energy a surface tension proportionality constant of γ = 0.005 kcal mol^−1^ Å^−2^ and a zero offset *b* were used.

### Calculation of the polar contribution to the solvation free energy

The polar contribution to the solvation free energy was determined using the PB approach (38) and applying the Adaptive Poisson-Boltzmann Solver (APBS) (39). The finite difference method in APBS was used for computing accurate solutions to the PB equation. The calculations were performed employing an automatically configured sequential focussing multigrid procedure. In this procedure, a less accurate solution on a coarse finite difference mesh covering the entire ribosome is used to define the boundary conditions for more accurate calculations with a finer discretization of the ligand binding site. The H50S structures were encapsulated in a cubic coarser grid with dimensions of 292 × 347 × 412 Å^3^ and a finer, final grid with dimensions of 25 × 12 × 15 Å^3^ focussed on the ligand. The electrostatic potential for the linezolid binding site in the H50S structures was obtained at a resolution of 0.19 Å.

The APBS calculations were performed after fitting the structures to a reference structure aligned to the principal axis. The principal axis alignment was done to ensure consistent grid enclosing of the complex for all snapshots. The continuum solvent dielectric constant (ε) was set to 80.0, and several solute dielectric constants ranging from 1 to 11 were tested. Preliminary tests revealed that a relatively high solute dielectric constant of 11 is best with respect to the rank ordering of different oxazolidinones binding to D50S and H50S, (14) likely because it accounts for the increased polarity of the oxazolidinone binding site (40). Using a higher dielectric constant is in line with other studies on the ribosome, (14,41) which implies that, compared to MM-PBSA calculations for proteins, (42,43) a higher solute dielectric constant might in general be required to investigate ligand-ribosome complex structures via the MM-PBSA approach. The dielectric boundary was defined by a 1.4 Å probe sphere. In all PB calculations, the PARSE parameter set (44) (radius of H = 1.0 Å, C = 1.7 Å, N = 1.5 Å, O = 1.4 Å, P = 2.0 Å) was used. A radius of 1.50 Å was assigned to Mg^2+^ ions. The calculations were performed with an ionic strength of 150 mM of monovalent ions and with an ion exclusion radius of 2 Å (45).

## RESULTS

### Overall structural stability of the linezolid-H50S complexes

**Figure 2.**
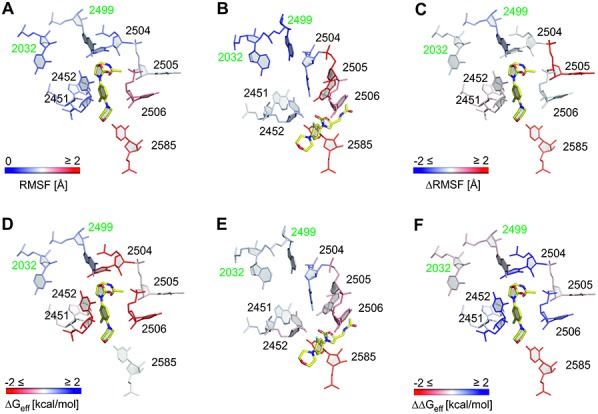
RMSF and per-nucleotide contributions to the effective binding energy. Shown are nucleotides of the first shell of the binding site along with the two mutation sites 2032 and 2499 investigated in this study. The structure with the smallest RMSD to the average structure of the last 20 ns of the respective MD trajectory was used for visualization; linezolid is colored in yellow. (**A**–**C**) Per-nucleobase RMSF obtained from MD simulations of linezolid-H50S_wt_ (A), linezolid-H50S_mut_ (B) (deep blue: RMSF 0 Å; white: 1 Å; deep red: RMSF ≥ 2 Å) as well as the difference (linezolid-H50S_mut_ – linezolid-H50S_wt_; deep blue: ≤ -2 Å; white: 0 Å; deep red: ≥ 2 Å). (**D–F**) Per-nucleotide contributions as computed by MM-PBSA for linezolid-H50S_wt_ (D), linezolid-H50S_mut_ (E) as well as the difference (linezolid-H50S_mut_ – linezolid-H50S_wt_) (F) (deep red: ≤ −2 kcal mol^−1^; white: 0 kcal mol^−1^; deep blue: ≥ +2 kcal mol^−1^). Except for the mutated nucleotides, the data for the per-nucleotide decomposition for linezolid-H50S_wt_ has been taken from (14).

All-atom explicit solvent MD simulations of 50 ns length each were performed for linezolid-H50S_wt_ (described in (14)) and linezolid-H50S_mut_, which constitute systems of the size of ∼8.5 * 10^5^ atoms. For investigating structural deviations along the trajectories, the RMSD of all or only ‘core’ residues were computed with respect to the starting structure for linezolid-H50S_mut_ (Supplementary Figure S3A; following (14) ‘core’ residues were selected by regarding only those residues with the 90% lowest RMSF of C_α_ and phosphorous atoms; here, the term ‘residue’ is used for both nucleotides and amino acids). Likewise, the RMSD of nucleotides forming the ligand binding site (first and second shell nucleotides of the PTC) (Supplementary Figure S3B) and the RMSD of linezolid (Supplementary Figure S3C) were computed after superpositioning the ‘core residues’. Comparing these values with those for linezolid-H50S_wt_ (14) shows that the structures of the large ribosomal subunits remain stable over the course of the trajectories, with RMSD values of the ‘core’ and binding site residues reaching plateaus of ∼2 Å. A major difference between both systems occurs with respect to the stability of the ligand binding mode: while the ligand in linezolid-H50S_wt_ shows RMSD values of ∼3 Å after 10 ns of MD simulations, which then remain stable (Supplementary Figure S3C), the RMSD of the ligand in linezolid-H50S_mut_ jumps to ∼6 Å after that time and then increases to values of up to 14 Å (Supplementary Figure S3C). Visual inspection of the linezolid-H50S_mut_ trajectory reveals that linezolid moves from the starting position (Figure 1B and C) further toward the A- and P-sites in the course of the simulation (Figure 2B). In 10 additional control simulations for linezolid-H50S_wt_ and linezolid-H50S_mut_, respectively, the binding mode of linezolid remains stable in eight cases when bound to H50S_wt_ (Supplementary Table S6 and Figure S7) and in four cases when bound to H50S_mut_ (Supplementary Table S7 and Figure S8). For this, the RMSD of linezolid and stacking interactions between the fluorophenyl ring of linezolid and nucleobases of A2451 and C2452 (see below) were evaluated. The difference in the relative proportions of bound linezolid (8/10 versus 4/10) is statistically significant (*P* < 0.05) according to a pairwise difference t-test. If, in addition, stacking interactions between the oxazolidinone core and the nucleobase of U2504 were evaluated (see below), the difference in the relative proportions of stable binding modes even increases (7/10 versus 2/10 for H50S_wt_ and H50S_mut_, respectively).

The observed unbinding events could be due to too cursory system preparation. This possibility can be highly likely ruled out because radezolid (6) and tedizolid (16), two novel oxazolidinone antibiotics that show activity against linezolid-resistant strains, (15) remain in the A-site binding region of H50S_mut_ in MD simulations of the same length (see below); the starting structures for these simulations were generated analogously to the one of linezolid-H50S_mut_. An inaccurate force field could also result in the linezolid unbinding. This possibility seems unlikely to us as well considering that all nucleotides in the first and second shell around linezolid are standard nucleotides (Supplementary Table S2) and, thus, are covered by the applied RNA force field (19). Furthermore, a quality analysis of force field parameters of GAFF (20) for linezolid showed good to very good agreement with quantum mechanical calculations (see above and in the Supplementary Data). Hence, the pronounced unbinding in the linezolid-H50S_mut_ case provides a first hint as to weakened binding interactions in the mutant. As a consequence, we only used the last 20 ns of both trajectories for comparative structural analyses.

### Effect of G2032A-C2499A mutations on structure and interaction network

The movement of linezolid in the H50S_mut_ structure is accompanied by conformational changes of nucleobases forming the first shell, especially U2504, G2505, U2506, A2451 and C2452 (Figure 2B) in comparison to the starting structure (Figure 1B). Most pronounced, the nucleobase of U2504 in H50S_mut_ moves to where the oxazolidinone core of linezolid was in the starting structure; likewise, G2505 moves to the starting location of the acetamide moiety. In contrast, only minor structural changes of both the ligand binding mode and the surrounding nucleobases have been observed for linezolid bound to H50S_wt_ (Figures 1B,C and 2A) (14).

**Figure 3.**
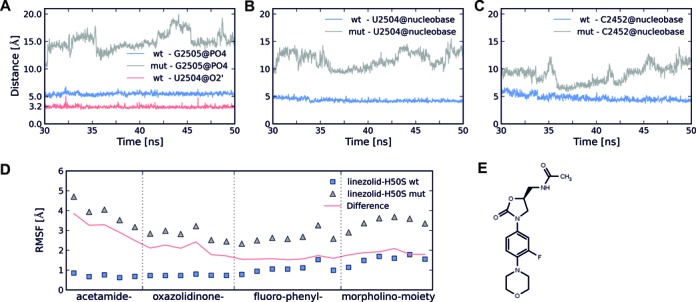
Interactions of linezolid with nucleotides of the first shell and RMSF of linezolid. (**A**) Distances monitoring hydrogen bond formation between linezolid's acetamide NH group and the oxygens of the phosphate group of G2505 (linezolid-H50S_wt_: blue, linezolid-H50S_mut_: gray; only the smallest distance found in each snapshot is plotted) and between linezolid's acetamide NH group and O2’ of U2504 (linezolid-H50S_wt_: red). (**B** and **C**): Distances monitoring aromatic stacking interactions between the centers of mass of the oxazolidinone core and the nucleobase of U2504 (B), and between the fluorophenyl ring and the nucleobase of C2452 (C). Distances for linezolid-H50S_wt_ and linezolid-H50S_mut_ simulations are depicted with blue and gray lines, respectively. (**D**) RMSF of linezolid atoms during the linezolid-H50S_wt_ (squares) and linezolid-H50S_mut_ (triangles) simulations. The red line represents the difference between the RMSF of linezolid-H50S_mut_ and linezolid-H50S_wt_ simulations. The data for the linezolid-H50S_wt_ simulation was taken from (14). (**E**) Chemical structure of linezolid.

We next investigated changes in the network of hydrogen bond and aromatic stacking interactions caused by the G2032A-C2499A double mutation. In the H50S co-crystal structure, (8) only one hydrogen bond interaction is formed between the acetamide NH group of linezolid and the phosphate group of G2505. This hydrogen bond is missing in the linezolid-D50S crystal structure, (7) indicating its weak nature. In addition, aromatic stacking interactions are formed in the H50S co-crystal structure between the oxazolidinone core and the nucleobase of U2504 as well as between the fluorophenyl ring and the nucleobase of C2452 (8). In the course of the linezolid-H50S_wt_ trajectory, these two aromatic stacking interactions remain stable (Figure 3B and C) whereas the hydrogen bond between linezolid's acetamide NH and G2505 breaks after 4 ns (data not shown) and does not re-form again (Figure 3A) (14). Instead, the ligand's acetamide NH group forms a strong hydrogen bond with the sugar part of U2504 (occupancy 75%; Figure 3A, cyan line) as a result of minor changes of the ligand binding mode. None of these interactions are present in the course of the linezolid-H50S_mut_ trajectory (Figure 3A–C) as expected from the large shift of linezolid described above. Together with C2452, A2451 forms the so-called A-site cleft (8). Monitoring aromatic stacking interactions between the fluorophenyl ring of linezolid and A2451 neither revealed stacking interactions in linezolid-H50S_wt_ nor in linezolid-H50S_mut_, however (data not shown).

Regarding interactions between nucleotides of the first to third shells, strong hydrogen bond interactions are formed in the linezolid-H50S_wt_ trajectory between nucleotides U2504 and C2452 (occupancy 93%), U2504 and U2500 (occupancy 67%), U2500 and C2452 (occupancy 88%), U2500 and A2055 (occupancy 60%), U2572 and G2032 (occupancies 61% and 66%), as well as between C2499 and A2453 (occupancy 99%) (Table 1, Supplementary Figure S4). Except for a hydrogen bond between U2504 and U2500 (occupancy 90%), all other hydrogen bonds are absent over the course of the linezolid-H50S_mut_ trajectory (Table 1, Supplementary Figure S4). Instead eight new hydrogen bonds are formed in the linezolid-H50S_mut_ trajectory, two between A2451 and G2447 (occupancy 73% and 75%), one between C2452 and A2451 (occupancy 60%), two between U2500 and A2453 (occupancies 82 and 99%), one between U2500 and G2447 (occupancy 73%), one between U2500 and A2032 (occupancy 87%), and one between G2505 and U2504 (occupancy 86%) (Table 1, Supplementary Figure S4).

**Table 1.**
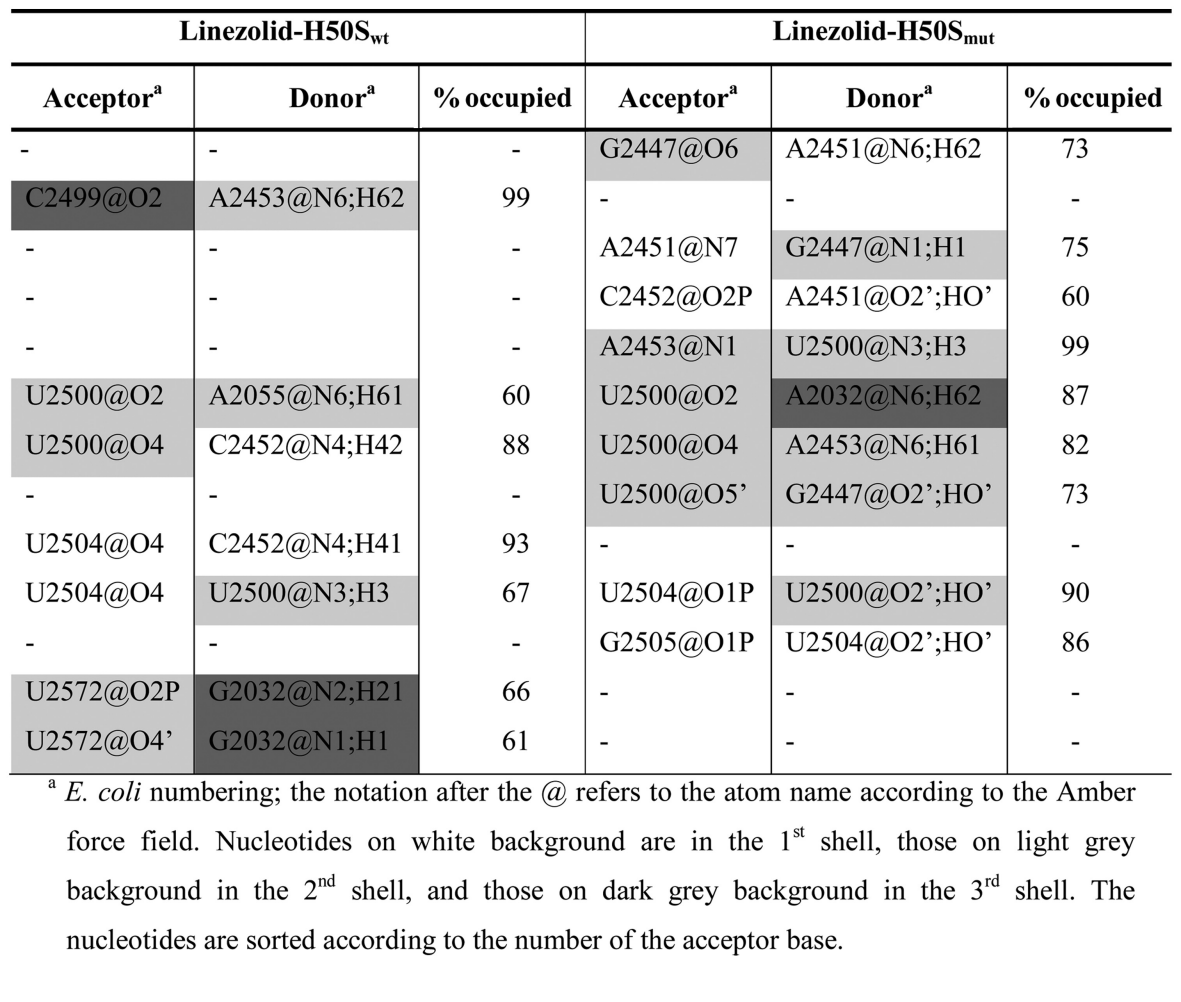
Hydrogen bonds with occupancy values >60%

Aromatic stacking interactions that occur only in the linezolid-H50S_wt_ trajectory were found between A2055 and U2504; in turn, stacking interactions between A2499 and U2500, A2032 and A2055, A2451 and C2452 as well as G2505 and U2506 occur only in the linezolid-H50S_mut_ trajectory (Table 2; Supplementary Figure S5).

**Table 2.**
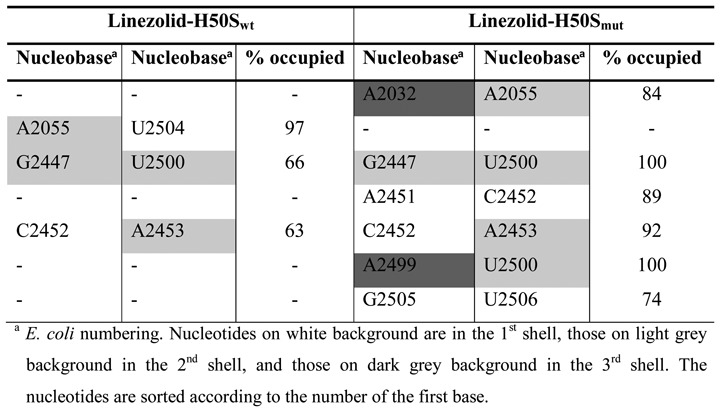
Aromatic stacking interactions with occupancy values >60%

In summary, major changes in the interaction network between first to third shell nucleotides are observed between linezolid-H50S_wt_ and linezolid-H50S_mut_ despite overall only moderate structural changes (RMSD ∼ 2 Å; see above).

### Effect of G2032A-C2499A mutations on the dynamics

Higher RMSF of nucleotides forming the first shell of the linezolid binding site (A2451, C2452, G2505, U2506, U2585; Figure 2A, B and Supplementary Table S3) were found in the linezolid-H50S_mut_ trajectory as compared to the linezolid-H50S_wt_ trajectory, with differences as large as 1.4 Å (U2585; Figure 2C). In contrast, nucleotides U2572 (second shell) and G2032 (third shell) were slightly more mobile in the linezolid-H50S_wt_ trajectory (differences < 0.4 Å). The higher RMSF of nucleotides A2451 and C2452 in linezolid-H50S_mut_ are surprising at first glance because these nucleotides make hydrogen bonds and stacking interactions with neighboring nucleotides in the linezolid-H50S_mut_ trajectory that do not occur in the linezolid-H50S_wt_ trajectory (see above; Tables 1 and 2). However, one needs to consider that linezolid moves away from its starting position in the linezolid-H50S_mut_ trajectory, which removes steric restrictions between the A-site cleft-forming nucleotides A2451 and C2452. In turn, the higher RMSF of G2032 in linezolid-H50S_wt_ can be explained in that G2032 only forms a hydrogen bond to U2572 there but a hydrogen bond to U2500 and stacking interactions to A2055 in linezolid-H50S_mut_ (Tables 1 and 2).

Regarding the ligand, the highest RMSF over the last 20 ns of the linezolid-H50S_wt_ trajectory are found in the region of the morpholino moiety (average over all atoms: 1.5 Å) whereas the acetamide, oxazolidinone and fluorophenyl moieties are less mobile (0.7, 0.7 and 1.1 Å (Figure 3D and E)) (14). This is in line with the analysis of stabilizing interactions, which indicated that linezolid's acetamide NH is involved in a hydrogen bond to U2504 (Figure 3A). In contrast, the shifted ligand in the linezolid-H50S_mut_ complex shows RMSF values as high as 4.7 and 3.7 Å at either end of the molecule (Figure 3D), suggesting that linezolid is not tightly bound at its new position.

### Effect of G2032A-C2499A mutations on per-nucleotide contributions to the effective binding energy

The above analyses were complemented by a structural decomposition (33) of MM-PBSA effective binding energies in order to investigate differences in the energetic contributions of nucleotides in the first and second shell of the PTC binding site (35,42). As in our previous study, (14) we pursued the single-trajectory approach (32) as it has proven to be a good and computationally more efficient approximation to the three-trajectory approach in ligand binding studies (32,46). The analysis was performed for the last 10 ns of the MD trajectories because the drift in effective binding energies (0.32 kcal mol^−1^ ns^−1^ for linezolid-H50S_wt_ (14); 3.62 kcal mol^−1^ ns^−1^ for linezolid-H50S_mut_) over time was lowest there for linezolid-H50S_wt_ (Supplementary Figure S6) (14). The much larger drift for linezolid-H50S_mut_ can be explained by the displacement of linezolid from its initial binding position further toward the A- and P-sites (Figure 2B). The difference in the total effective binding energy of linezolid in linezolid-H50S_mut_ versus linezolid-H50S_wt_ is 7.18 ± 0.77 kcal mol^−1^ (mean ± SEM determined over 500 snapshots extracted in intervals of 20 ps; Supplementary Table S4). The sign of the difference agrees with experimental results according to which *M. smegmatis* revealed a minimum inhibitory concentration (MIC) of linezolid of 2 μg ml^−1^ for the wild-type (SZ558 strain) and a MIC of 8 μg ml^−1^ for the G2032A-C2499A mutant (10). The positive total effective binding energy of 7.38 ± 0.74 kcal mol^−1^ for linezolid binding to H50S_mut_ (Supplementary Table S4) also reflects that the initial binding mode of linezolid in H50S_mut_ is significantly less stable (Figure 2B, Supplementary Tables S6 and S7, Figures S7 and S8).

As to a quantitative comparison, the computed difference in the total effective binding energy seems to exceed the difference in the binding free energy inferred from the MIC (at *T* = 300 K: ∼0.8 kcal mol^−1^) by ∼9-fold. We note, however, that MIC characterizes the lowest concentration of an antibiotic that will inhibit the visible growth of a microorganism after some incubation (47) and as such is generally regarded as the most basic measurement of the activity of an antibiotic against an organism (48). Several examples for the lack of direct, quantitative correlations between antibiotic binding and the antibiotic sensitivity of the corresponding organism have been noted (e.g. see (49) with respect to penicillin binding). Thus, ideally, our results should be compared to biophysical binding data obtained for *H. marismortui* ribosomes; however, to the best of our knowledge, no such data is available. An inappropriate computational model could be another reason for the variance between the computed difference in the total effective binding energy and the inferred difference in the binding free energy from the MIC. While we cannot exclude this possibility, it appears unlikely to us given that in our recent study on linezolid binding to H50S and D50S, employing the same computational model, the computed ratio of the association constants agreed to within a factor of 100 with the ratio of concentrations required for a successful co-crystallization of linezolid in H50S or D50S (14,50). Our previous results were also in line with results from functional assays on *S. aureus* and *H. marismortui* ribosomes where a selectivity of linezolid toward the eubacterial ribosome was found (6,14). Finally, the movement of linezolid from the starting position further toward the A- and P-sites (see above) might result in a linezolid configuration in H50S_mut_ that is still inhibitory and so explain the small change in the MIC between wild-type and G2032A-C2499A mutant of *M. smegmatis*. In fact, the position occupied by linezolid in H50S_mut_ after the movement overlaps with the binding site of sparsomycin and dalfopristin (2). However, much longer MD simulations are required to test if the new binding mode of linezolid is stable.

At a per-nucleotide level, C2452 and U2504 show the largest (>2.9 kcal mol^−1^) differences in their contributions to the effective binding energy when comparing linezolid-H50S_mut_ versus linezolid-H50S_wt_ (Figure 2D–F; Supplementary Table S5; the SEM in the difference in the effective binding energy due to one nucleotide is assumed to be of a similar magnitude than the one for the overall difference (see above) (14)). Of all first shell nucleotides, these nucleotides are closest to the mutation sites in the third shell. U2585 also shows a large difference but of opposite sign (−2.9 kcal mol^−1^), in line with the fact that this nucleotide interacts favorably with the shifted linezolid in H50S_mut_ (Figure 2E). In all, the nucleotides of the first shell contribute almost 90% to the difference in the total effective binding energy (Supplementary Table S5). In contrast, the second shell contribution is small and even in favor of binding to the mutant (Supplementary Table S5). These findings are in line with results from our previous study (14) on the proportion of contributions of first and second shell nucleotides to the total effective binding energies *per se*. Finally, the contributions of the nucleotides at the mutations sites 2032 and 2499 differ between H50S_mut_ versus H50S_wt_ by −0.31 and −0.27 kcal mol^−1^ (Supplementary Table S5).

### Effect of G2032A-C2499A mutations on the binding of novel oxazolidinone antibiotics

Radezolid (6) and tedizolid (16) are two novel oxazolidinone antibiotics that have completed two phase-II clinical trials (Safety and efficacy study of oxazolidinone, 2014. https://www.clinicaltrials.gov/ct2/results?term=radezolid&Search=Search) or have been approved by the FDA (FDA approves Sivextro to treat skin infections, 2014. http://www.fda.gov/NewsEvents/Newsroom/PressAnnouncements/ucm402174.htm), respectively. Both antibiotics show activity against linezolid-resistant strains (15). Hence, it is interesting to investigate to what extent they are affected by the G2032A-C2499A mutations. We thus performed all-atom explicit solvent MD simulations of 50 ns length each for radezolid-H50S_mut_ and tedizolid-H50S_mut_. In the course of both simulations, the binding position of the ligand remained unaffected by the double mutation as demonstrated by almost constant distances between radezolid's acetamide group / tedizolid's hydroxyl group and G2505, the oxazolidinone core and U2504, and the fluorophenyl ring and C2452 (Supplementary Figure S9A–C). Furthermore, the RMSD of radezolid and tedizolid in H50S_mut_ of ∼3.5 Å (Supplementary Figure S9E) compares favorably to that of linezolid in H50S_wt_ (Supplementary Figure S3C). Note that, in contrast to the linezolid-H50S_wt_ MD simulation, the simulations of radezolid-H50S_mut_ and tedizolid-H50S_mut_ were started from modeled complex structures due to the lack of appropriate crystal structures.

## DISCUSSION

This study reveals at an atomistic level how the G2032A-C2499A double mutation in the third shell of the H50S A-site confers linezolid resistance by a complex set of effects that percolate to the antibiotic binding site. From a global point of view, the long-distance effect is markedly manifested by an instable binding mode of linezolid in H50S_mut_ observed already after 10 ns of MD simulations (Supplementary Figure S3C), which is in contrast to stable linezolid binding modes over 50 ns of MD simulations observed in H50S_wt_ (Supplementary Figure S3C) and D50S (see our previous study (14)). This finding is confirmed by 10 control simulations of linezolid-H50S_wt_ and linezolid-H50S_mut_, respectively, where the difference in the relative proportions of stable binding modes is statistically significant in favor of H50S_wt_. The observed linezolid displacement in H50S_mut_ is accompanied by a positive total effective binding energy (Supplementary Table S4) and high RMSF values of the ligand at the new position (Figure 3D), suggesting that the ligand is not tightly bound at the new position and that a further displacement ought to be expected if the MD simulations were elongated.

**Figure 4.**
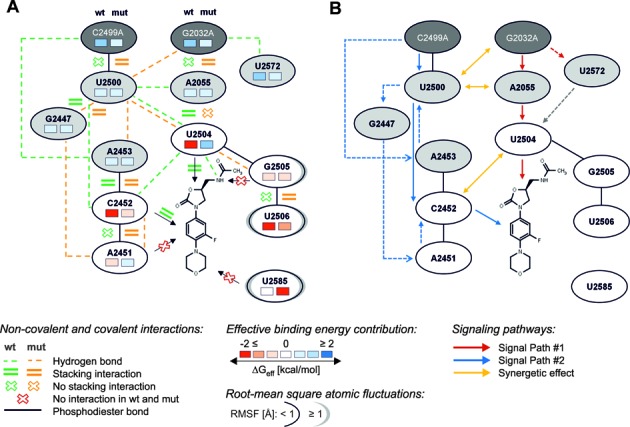
Summary of interactions, contributions to the effective binding energy, and RMSF as well as proposed signaling pathways from the mutation sites to the binding site. (**A**) Scheme summarizing non-covalent interactions (Tables 1 and 2), contributions to the effective binding energy (Figure 2), and RMSF (Figure 2) of nucleotides forming the first (white ellipses) and second (light gray ellipses) shell of the PTC binding site and the two mutation sites (dark gray ellipses). Non-covalent interactions are displayed in green for linezolid-H50S_wt_ and in orange for linezolid-H50S_mut_. Information on contributions to the effective binding energy and RMSF of each nucleotide are depicted on the left side for linezolid-H50S_wt_ and on the right side for linezolid-H50S_mut_, respectively. See the legend for further details. (**B**) Routes by which the information of the double mutation is transmitted via second shell nucleotides to U2504 (red arrows) and C2452 (blue arrows), respectively, as well as synergistic effects between these two routes (yellow arrows). The gray arrow depicts a blocking effect of U2504 by U2572 in H50S_wt_ described in (9), but not observed on the time scale of our simulations.

At a local level, the effect of the double mutation is summarized in Figure 4. Regarding critical interactions, the binding mode of linezolid in H50S_wt_ is stabilized by a hydrogen bond between the antibiotic's acetamide group and the sugar part of U2504 (Figure 3A) or between the acetamide group and the phosphate group of G2505 (Supplementary Table S6) as observed in the crystal structure (8). In addition, stacking interactions between linezolid's fluorophenyl ring and C2452 (Figure 3C), in agreement with the crystal structure, (8) and between the oxazolidinone core and U2504 (Figure 3B) occur. Notably, all these interactions are amiss in linezolid-H50S_mut_, most likely as a result of the pronounced conformational changes observed for the respective nucleobases with respect to the starting structure. In agreement, the two nucleotides C2452 and U2504 also show the most disfavorable relative contributions to the effective binding energy at a per-nucleotide level (Figure 2F, Supplementary Table S5). In all, our structural and energetic analyses identify U2504 and C2452 as spearheads among the first shell nucleotides that exert the most immediate effect on linezolid binding due to the remote double mutation. For U2504, which also has a prominent role in determining the selectivity of antibiotics binding to the A-site, (9,51) a pivotal role in resistance to linezolid via mechanisms by which the nucleotide is perturbed by proximal mutations has been suggested previously based on comparative crystallographic studies (9). In contrast, aside from a direct mutation, (52) C2452 has not yet been linked to linezolid resistance resulting from remote mutations. In this context it is interesting to note that oxazolidinone derivatives that show activity against linezolid-resistant strains and have undergone advanced clinical evaluations or have recently been approved by the FDA, such as radezolid (6) or tedizolid (16), have been extended at the morpholino end of linezolid (Supplementary Figure S9F), i.e. at that end of the molecule that should be least influenced by the here investigated mutations 2499 and 2032. In contrast to linezolid, these two oxazolidinone antibiotics remain in the A-site binding region in H50S_mut_ during our MD simulations (Supplementary Figure S9), suggesting that the extensions compensate for the influence of the mutations.

What leads to C2452 and U2504 being spearheads? The structural decomposition of the difference in effective binding energy (Figure 2F, Supplementary Table S5, Figure 4A) reveals only minor changes at the per-nucleotide level for nucleotides of the second shell and the mutation sites C/A2499 and G/A2032, which are mostly slightly in favor of the H50S_mut_ structure. Note that this result must be interpreted with caution because in the single-trajectory MM-PBSA approach pursued here conformational changes of receptor and ligand upon complex formation are ignored (32). Still, it leads to the interesting suggestions that either the effect of the double mutation does not result in gross structural reorganizations in the second and third shell, and hence no changes in the effective energy are associated with them, or structural reorganizations are associated with mutually compensating changes in the effective energy. Our analyses of structural changes and changes in the interaction network indicate that the latter applies (Figure 4A): As to hydrogen bonds second and third shell nucleotides are involved in, five are lost in H50S_mut_ compared to H50S_wt_ (C/A2499^…^A2453; U2500^…^A2055; U2500^…^C2452; U2572^…^G/A2032 (2x)) and six are formed (G2447^…^A2451 (2x); U2500^…^G2447; A2453^…^U2500 (2x); U2500^…^A2032); this is accompanied by two stacking interactions involving second and third shell nucleotides formed in H50S_mut_ compared to H50S_wt_ (G/A2032^…^A2055; C/A2499^…^U2500) and one lost (A2055^…^U2504). In line with the balanced numbers of lost and newly formed interactions, the mobility of second and third shell nucleotides only changes marginally between H50S_wt_ and H50S_mut_ (Figure 4A; Supplementary Table S3).

From this complex reorganization in the network of inter-nucleotide interactions, we can suggest two main routes by which the information of the double mutation is transmitted via second shell nucleotides to U2504 and C2452 (Figure 4B):

(i) The G2032A mutation results in the formation of base stacking interactions with A2055, which likely contributes to the loss of stacking interactions of A2055 with U2504, which in turn releases a restraint on the conformation of the U2504 base (Figure 4B; red arrows). Nucleotide 2055 has been described before to have a prominent role in influencing the conformation of the U2504 base in eubacterial versus archaeal/eukaryotic ribosomes (9,14). Furthermore, the G2032A mutation leads to a loss of a hydrogen bond with U2572 (Figure 4B; red dashed arrow). U2572 in H50S_wt_ has been described to block the U2504 ribose from shifting away from the PTC (Figures 1B and 2A); (9) the lost hydrogen bond may thus release this blocking effect (Figure 4B; gray dashed arrow) although we were unable to observe this on the time scale of our simulations. These findings can explain how the G2032A mutation directly perturbs the conformation of U2504. This result strongly supports the hypothesis that U2504 is important for binding of PTC antibiotics and that its conformation is maintained and restrained, among other second shell nucleotides, by nucleotides 2055 and 2572, which was previously derived based on the comparative analysis of ribosomal crystal structures (9).

(ii) In contrast, the C2499A mutation directly perturbs C2452 by way of two subroutes: (a) As an immediate effect, the mutation leads to the formation of stacking interactions with U2500, which changes the orientation of the latter base such that a hydrogen bond to C2452 is broken (Figure 4B; blue arrows). (b) More indirectly, a hydrogen bond is lost due to the mutation between A2499 and A2453. Because of this and the change in the orientation of U2500 (see (a)) hydrogen bonds between A2453 and U2500 as well as U2500 and G2447 are formed. The latter leads to G2447 taking up a new orientation, which allows it to form a hydrogen bond with A2451. Finally, A2451 then forms stacking interactions to C2452 (Figure 4B; blue dashed arrows). Together with (a) this leads to a change in the orientation of C2452 such that the stacking interactions with linezolid are lost. To the best of our knowledge, such an indirect perturbation of C2452 by C2499A has not yet been described.

Although an example for a G2032A (C2499A) mutation without the involvement of 2499 (2032) has been described, leading to linezolid resistance in *E. coli* (53) (*Halobacterium halobium* (52)), usually a G2032A mutation is accompanied by a C2499A mutation (54). This can be rationalized by the finding of synergistic effects on antibiotic susceptibilities due to the double mutation, (10) and it has been suggested based on the comparative analysis of ribosomal crystal structures that favorable polar attractions between A2499 and A2032 stabilize the latter nucleotide (9). However, we do not find any hydrogen bonds between these two nucleotides in the course of the MD simulation of H50S_mut_ (data not shown). What then leads to the observed synergy? Our structural analysis suggests that there is cross-talk between the two main routes that transmits information (i) from the mutation site G2032A via U2500 to C2452, (ii) from the C2499A mutation site via U2500 and A2055 to U2504 and (iii) from U2504 to C2452 and vice versa (Figure 4B, yellow arrows): (i) the G2032A mutation leads to the formation of a hydrogen bond with U2500; (ii) the C2499A mutation leads to stacking interactions in H50S_mut_ with U2500 that fosters the fixation of U2500 in a conformation not competent to form a hydrogen bond to A2055; (iii) the hydrogen bond between C2452 and U2504 is lost in H50S_mut_. These findings suggest that synergistic effects between the two mutations arise from an indirect manner rather than from direct interactions between the mutated nucleotides. However, additional comparisons to MD simulations of linzelid-H50S complex structures with the respective single mutations will be required to provide direct evidence for this.

To the best of our knowledge, this is the first study to investigate resistance to antibiotics binding to the A-site of the 50S ribosomal subunit due to remote mutations by MD simulations and free energy calculations, thus considering aspects of structure, dynamics, and energetics simultaneously. We chose the linezolid-H50S structure (8) as a model for several reasons: (i) This structure has been solved at a resolution of 2.7 Å, (8) which is the highest resolution available for complex structures of linezolid bound to the large ribosomal subunit, and the structure was successfully used in the previous computational study by us (14). (ii) The *archaeal* H50S subunit shows typical *eubacterial* elements at the PTC in that the linezolid-bound conformation of U2504 is nearly identical to that of the *apo* conformation of the homologous nucleotide in bacterial ribosomes, which can explain why H50S binds oxazolidone antibiotics (7,51). (iii) The H50S subunit possesses *eukaryotic* elements in the second shell PTC nucleotides (12) (Supplementary Table S2), which can explain why archaeal ribosomes are generally considered more ‘eukaryotic-like’ with respect to their antibiotic specificities (7). The H50S subunit can thus be regarded as an intermediate, which may be particularly suited for investigating effects of nucleotide exchanges at remote sites on linezolid binding, where low sequence conservations have been observed between eukaryotes and bacteria and which have been associated with species selectivity of binding and resistance in bacteria. Previously, major insights into the structural basis for (cross-)resistance have been obtained by comparative analysis of complex structures of ribosomes bound to PTC antibiotics (9–12). However, these studies did not compare ribosome structures mutated at positions 2032 or 2499 to the respective wild-type structures. Rather, for remote mutations, the analyses suggested contributions of mutated nucleotides to resistance deduced from observations of discrete nucleotide conformations across different species. A recent study by us on the determinants of the species selectivity of oxazolidinone antibiotics suggested that analyses based on static crystal structures and qualitative arguments on interactions may not reach far enough in this case (14).

As to the implications of our work, the question arises to what extent our results are transferable between species. In our view, one needs to exercise caution in this context given that A2055 in H50S differs from C2055 usually found in bacterial ribosomes and considering the important role of nucleotide 2055 in restraining the conformation of U2504 (see above). This view is corroborated by experimental findings according to which the single mutation G2032A confers resistance to linezolid in *E. coli* (53) but neither in *T. thermophilus* (55) nor in *M*. *smegmatis*, (10) demonstrating organism-dependent effects of the mutation even within a series of bacterial ribosomes. Another question relates to the predictability of cross-resistances from our work. The marked conformational change of U2504 observed in the MD simulations of H50S_mut_ together with this nucleotide's central role in the overlapping binding modes of linezolid, (8) chloramphenicol, (56) and valnemulin (inferred from the binding mode of the related pleuromutilin tiamulin (57)) may rationalize why the G2032A-C2499A double mutation in *M. smegmatis* results in reduced antibiotics susceptibilities in all three cases (10). However, our findings do not allow to explain why the susceptibility to clindamycin, the binding mode of which overlaps with the ones of the other three antibiotics, (56) is uninfluenced by the double mutation in *M. smegmatis* (10). Apparently, there is no simple relationship between overlapping binding modes and cross-resistance. Additional (and possibly synergistic) effects must be considered, such as found in terms of the influence of C2452 on linezolid binding in our case. In our view, this makes the prediction of cross-resistance without explicitly considering the respective mutation and the potentially influenced antibiotic difficult.

Our analyses of structural, dynamic and energetic determinants reveal how remote mutations exert an influence on the susceptibility of a PTC antibiotic. The determinants are consistent in describing effects of a complex but balanced reorganization in the network of inter-nucleotide interactions that percolates from the mutation sites to the PTC. In particular, identifying cross-talk between the two main routes of information transfer, which could explain the experimentally observed synergy of the double mutation, goes beyond current knowledge on the structural basis for (cross-)resistance. The possibility to extrapolate our results to other organisms and/or resistances to other antibiotics is limited due to the complexity of the involved effects. Yet, as demonstrated in this work, it has become possible to explicitly investigate the respective combination of organism/mutation/antibiotic within the time range available by current state-of-the art MD simulations.

## Supplementary Material

SUPPLEMENTARY DATA
