# Supplementary material for: Complex long-distance effects of mutations that confer linezolid resistance in the large ribosomal subunit
Source: Nucleic Acids Res. 2015 Jul 21;43(16):7731–43. doi: 10.1093/nar/gkv729 (PMC4652758; doi:10.1093/nar/gkv729)
Supplement: SUPPLEMENTARY DATA [file supp_43_16_7731__index.html]

Complex long-distance effects of mutations that confer linezolid resistance in the large ribosomal subunit — Complex long-distance effects of mutations that confer linezolid resistance in the large ribosomal subunit — SUPPLEMENTARY DATA 

# Complex long-distance effects of mutations that confer linezolid resistance in the large ribosomal subunit

## SUPPLEMENTARY DATA

- SUPPLEMENTARY DATA
