# Supplementary material for: Complex long-distance effects of mutations that confer linezolid resistance in the large ribosomal subunit
Source: Nucleic Acids Res. 2015 Jul 21;43(16):7731–43. doi: 10.1093/nar/gkv729 (PMC4652758; doi:10.1093/nar/gkv729)
Supplement: SUPPLEMENTARY DATA [file supp_gkv729_nar-00083-r-2015-File006.pdf]

SUPPLEMENTARY DATA

Complex long-distance effects of mutations that confer linezolid resistance in the large ribosomal subunit

Simone Fulle[§$], Jagmohan S. Saini[$], Nadine Homeyer, Holger Gohlke*

Institute for Pharmaceutical and Medicinal Chemistry, Department of Mathematics and Natural Sciences, Heinrich-Heine-University, Universitätsstr. 1, 40225 Düsseldorf, Germany

* Corresponding author: Universitätsstr. 1, 40225 Düsseldorf, Germany.
Phone: (+49) 211 81-13662. Fax: (+49) 211 81-13847. E-mail: gohlke@uni-duesseldorf.de.

[§] Present address: BioMed X Innovation Center, Im Neuenheimer Feld 583, 69120 Heidelberg, Germany.

[$] Joint first authors.

Quality analysis of force field parameters for linezolid

In order to investigate the quality of the force field parameters used in the MD simulations for the oxazolidinone antibiotic linezolid, structures of this antibiotic optimized at the molecular mechanical and the quantum mechanical level were compared. The optimization at the molecular mechanical level consisted of 200 steps conjugate gradient minimization with *sander* (1) applying the same parameters as in the H50S-linezolid MD simulations. The optimization at the Hartree-Fock (HF) level was conducted with the 6-31G* basis set using Gaussian09 (2). Comparison of the optimized structures yielded root mean-square deviations of 0.019 Å and 1.6° for bond lengths and angles, respectively.

In addition, energy profiles calculated at the molecular mechanical or quantum mechanical level of selected torsion angles of linezolid (Figure S1) were compared. For this, substructures of linezolid in which the respective torsion angle was changed (and constrained) in intervals of 5° were optimized at the HF/6-31G* level using Gaussian09 (2). The resulting energies were compared to those obtained at the molecular mechanical level, applying parameters for the fragments according to those of the H50S-linezolid MD simulations. Charges of atoms of the substructures that were not present in linezolid were chosen such that the overall molecular fragments were neutral. For torsion angles C13-N3-C12-C9 ($r^2 = 0.86$; see torsion profiles in Figure S2), O2-C4-C3-N1 ($r^2 = 0.84$), C6-C4-C3-N1 ($r^2 = 0.95$), and C4-C3-N1-C2 ($r^2 = 0.97$) good correlations were found between the quantum mechanical and molecular mechanical energy profiles. For the C8-C7-N2-C5 torsion the molecular mechanics energy profile was considerably affected by large repulsive van der Waals interactions in the quantum mechanically optimized structures at 0° and 180°. However, the quantum mechanical and the dihedral energy profiles were well correlated, too ($r^2 = 0.89$).

Supplementary Tables

Table S1: Nucleotides mediating linezolid resistance (Table adapted from ref. (3)).

E. coli [a,b]	***D. radiodurans*** [c]	***H. marismortui*** [c]
2032	2015	2073
2062	2045	2103
2447	2426	2482
2452	2431	2487
2453	2432	2488
2499	2478	2534
2500	2479	2535
2503	2482	2538
2504	2483	2539
2505	2484	2540
2576	2555	2611

[a] *E. coli* numbering
[b] From ref. (3)
[c] Taken from http://www.riboworld.com/nuctrans/

Table S2: Nucleotide differences in the first and second shell of the linezolid binding site of bacterial, archaeal, and eukaryotic ribosomes.[a]

**Nucleotides**	**Bacteria**				**Archaea**	**Eukarya**
	E. coli[a]	***D. radiodurans***[b]	***T. thermophilus***[b]	***M. smegmatis***[c]	***H. marismortui***[b]	***S. cerevisiae***[b]
First-shell	A2451	A	A	A	A	A
	C2452	C	C	C	C	C
	U2504	U	U	U	U	U
	G2505	G	G	G	G	G
	U2506	U	U	U	U	U
	U2585	U	U	U	U	U
Second-shell	C2055	**C**	**C**	**C**	**A**	**A**
	G2447	G	G	G	G	G
	A2453	**A**	**A**	**A**	**A**	**U**
	U2500	U	U	U	U	U
	A2572	A	A	A	**U**	A

[a] *E. coli* numbering.
[b] Used PDB codes: *D. radiodurans* (3DLL), *T. thermophilus* (2J01), *H. marismortui* (3CPW), and *S. cerevisiae* (3U5D).
[c] Taken from ref. (4).

Table S3: Root-mean square atomic fluctuations (RMSF) on a per-nucleotide and on a per-nucleobase level for linezolid-H50S$_{wt}$ and linezolid-H50S$_{mut}$.

**Nucleotide no.**[a]	**Linezolid-H50S$_{wt}$**[b]		**Linezolid-H50S$_{mut}$**[b]		**Δ H50S$_{mut}$-H50S$_{wt}$**[b]	
First shell	Per-nucleotide	Per-nucleobase	Per-nucleotide	Per-nucleobase	Per-nucleotide	Per-nucleobase
A2451	0.69	0.69	1.13	0.98	0.44	0.29
C2452	0.79	0.60	0.89	0.83	0.10	0.23
U2504	0.55	0.57	0.51	0.61	-0.04	0.04
G2505	0.68	0.90	1.53	2.66	0.85	1.76
U2506	0.91	1.42	1.10	1.36	0.19	-0.06
U2585	1.27	1.85	2.67	3.74	1.40	1.89
Second shell						
A2055	0.52	0.49	0.44	0.49	-0.08	0.00
G2447	0.44	0.44	0.40	0.41	-0.04	-0.03
A2453	0.61	0.49	0.59	0.45	-0.02	-0.04
U2500	0.40	0.43	0.35	0.39	-0.05	-0.04
U2572	0.71	0.97	0.47	0.45	-0.24	-0.52
Third shell						
G/A2032	0.81	0.85	0.49	0.41	-0.32	-0.44
C/A2449	0.48	0.53	0.41	0.45	-0.07	-0.08

[a] *E. coli* numbering.
[b] In Å.

Table S4: Components of the effective energy for linezolid binding to H50S.[a]

**Contribution**[b]	**Linezolid-H50S$_{wt}$**		**Linezolid-H50S$_{mut}$**		**Δ H50S$_{mut}$ – H50S$_{wt}$**	
	**Mean**[c]	**σ**[d]	**Mean**[c]	**σ**[d]	**Mean**[c]	**σ**[d]
ΔH_{elec}	-2.96	0.03	1.34	0.10	4.30	0.10
ΔH_{vdW}	-40.56	0.14	-32.77	0.21	7.79	0.25
ΔH_{gas}	-43.52	0.14	-31.43	0.17	12.09	0.22
ΔG_{PB}	47.33	0.17	42.36	0.61	-4.97	0.63
$\Delta G_{nonpolar}$	-3.61	0.01	-3.55	0.02	0.06	0.02
$\Delta G_{effective}$	0.20	0.20	7.38	0.74	7.18	0.77

[a] Gas phase and solvation free energy contributions were determined by the MM-PBSA approach, considering 500 snapshots from the last 10 ns of MD simulations of the linezolid-H50S complexes.
[b] H_{elec} : electrostatic energy; H_{vdW} : van der Waals energy; H_{gas} : gas phase energy; G_{PB} : polar part of the solvation free energy; $G_{nonpolar}$: non-polar part of the solvation free energy; $G_{effective}$: effective energy.
[c] Mean contributions in kcal mol^{-1}.
[d] Standard error in the mean values in kcal mol^{-1}.

Table S5: Effective binding energy contributions between linezolid-$H50S_{mut}$ and linezolid-$H50S_{wt}$ on a per-nucleotide level.[a]

**Nucleotide no.**[b]	**Linezolid-$H50S_{wt}$**[f]	**Linezolid-$H50S_{mut}$**[g]	**Δ^h $H50S_{mut}$[g] – $H50S_{wt}$[f]**
A2451[c]	-0.12	0.14	0.26
C2452[c]	-5.54	-0.29	5.25
U2504[c]	-2.41	0.54	2.95
G2505[c]	-0.19	-0.41	-0.22
U2506[c]	-1.79	-0.80	0.99
U2585[c]	0.02	-2.84	-2.86
Σ first shell			6.37
A2055[d]	0.29	0.16	-0.13
G2447[d]	0.17	0.30	0.13
A2453[d]	0.23	0.32	0.09
U2500[d]	0.11	0.20	0.09
U2572[d]	0.80	0.27	-0.53
Σ second shell			-0.35
G/A2032[e]	0.44	0.13	-0.31
C/A2499[e]	0.46	0.19	-0.27
Σ third shell			-0.58

[a] Effective binding energies for first and second shell nucleotides of the ligand binding site were computed by the MM-PBSA approach considering 500 snapshots from the last 10 ns of MD simulations of the linezolid-H50S complexes. In kcal mol^{-1}. The standard error of the mean (SEM) varies between 0.001 and 0.046 kcal mol^{-1}.

[b] Nucleotide number according to *E. coli* numbering.

[c] Nucleotides of the first shell of the ligand binding site.

[d] Nucleotides of the second shell of the ligand binding site.

[e] Nucleotides of the third shell of the ligand binding site.

[f] Wild type H50S in complex with linezolid.

[g] H50S with G2032A-C2499A double mutation in complex with linezolid.

[h] Difference between mean effective binding energies for linezolid-$H50S_{mut}$ and linezolid-$H50S_{wt}$.

Table S6: Summary of the structural analysis of linezolid-H50S$_{wt}$ control simulations.[a]

Trajectory no.	RMSD [b]	Stacking interaction [c]		Overall stability [d]
		Oxazolidinone core	Fluoro-phenyl ring	
prod_01	Stable	Stable	Stable	Stable
prod_02	20 ns	20 ns	2 ns	*Instable*
prod_03	Stable	Stable	Stable	Stable
prod_04	Stable	Stable	Stable	Stable
prod_05	Stable	2 ns	Stable	Stable
prod_06	Stable	Stable	Stable	Stable
prod_07	Stable	Stable	Stable	Stable
prod_08	Stable	Stable	Stable	Stable
prod_09	Stable	Stable	Stable	Stable
prod_10	5 ns	35 ns	38 ns	*Instable*

[a] In three of the trajectories, stable hydrogen bonds were formed either between linezolid's acetamide NH group and the oxygens of the phosphate group of G2505 (as present in the X-ray structure; prod_06) or with O2' of U2504 (as present in the initial linezolid-H50S$_{wt}$ trajectory; prod_04 and prod_07). Hydrogen bonds were defined by a distance cutoff of 3.2 Å and an angle cutoff of 120° and were considered stable if their occupancies attained > 60 % (percent of simulation time in which the hydrogen bond is formed) during the last 20 ns of the trajectory.
[b] RMSD values of linezolid with respect to the starting structure were considered stable if they stayed below 4 Å during the last 10 ns of the respective trajectory (Figure S7). Otherwise, a time point of the MD simulation is provided when the RMSD starts exceeding 4 Å.
[c] Stacking interactions between the oxazolidinone core or the fluoro-phenyl ring and the nucleobase of U2504 or A2451/C2452, respectively, were defined by a distance cutoff of 5.0 Å from one ring center to another and considered stable if their occupancies attained > 60 % during the last 20 ns of the trajectory. Otherwise, a time point of the MD simulation is provided when the stacking interaction ceases to exist. In the case of stacking interactions with the fluoro-phenyl ring, the smallest distance to the nucleobases of A2451 and C2452 was considered, respectively.
[d] The binding mode was considered stable if the RMSD values and the stacking interaction with the fluoro-phenyl ring were stable.

Table S7: Summary of the structural analysis of linezolid-H50S$_{mut}$ control simulations.[a]

**Trajectory no.**	**RMSD** [b]	**Stacking interaction** [c]		**Overall stability** [d]
		Oxazolidinone core	Fluoro-phenyl ring	
prod_01	Stable	Stable	2 ns	*Instable*
prod_02	Stable	Stable	Stable	Stable
prod_03	Stable	10 ns	Stable	Stable
prod_04	5 ns	5 ns	5 ns	*Instable*
prod_05	20 ns	30 ns	Stable	*Instable*
prod_06	Stable	Stable	Stable	Stable
prod_07	Stable	30 ns	Stable	Stable
prod_08	35 ns	37 ns	Stable	*Instable*
prod_09	1 ns	1 ns	Stable	*Instable*
prod_10	25 ns	25 ns	5 ns	*Instable*

[a] In none of the trajectories were stable hydrogen bonds formed between linezolid's acetamide NH group and the oxygens of the phosphate group of G2505 (as present in the X-ray structure) or with O2' of U2504 (as present in the linezolid-H50S$_{wt}$ trajectory). Hydrogen bonds were defined by a distance cutoff of 3.2 Å and an angle cutoff of 120° and were considered stable if their occupancies attained > 60 % (percent of simulation time in which the hydrogen bond is formed) during the last 20 ns of the trajectory.

[b] RMSD values of linezolid with respect to the starting structure were considered stable if they stayed below 4 Å during the last 10 ns of the respective trajectory (Figure S7). Otherwise, a time point of the MD simulation is provided when the RMSD starts exceeding 4 Å.

[c] Stacking interactions between the oxazolidinone core or the fluoro-phenyl ring and the nucleobase of U2504 or A2451/C2452, respectively, were defined by a distance cutoff of 5.0 Å from one ring center to another and considered stable if their occupancies attained > 60 % during the last 20 ns of the trajectory. Otherwise, a time point of the MD simulation is provided when the stacking interaction ceases to exist. In the case of stacking interactions with the fluoro-phenyl ring, the smallest distance to the nucleobases of A2451 and C2452 was considered, respectively.

[d] The binding mode was considered stable if the RMSD values and the stacking interaction with the fluoro-phenyl ring were stable.

Supplementary Figures

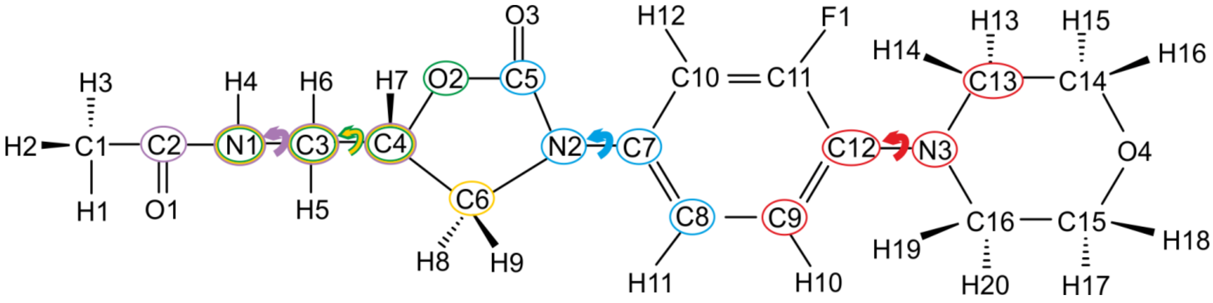

Figure S1. Schematic representation of linezolid. Atoms of studied torsion angles are marked by colored cycles, and bonds around which rotation occurs are indicated by arrows.

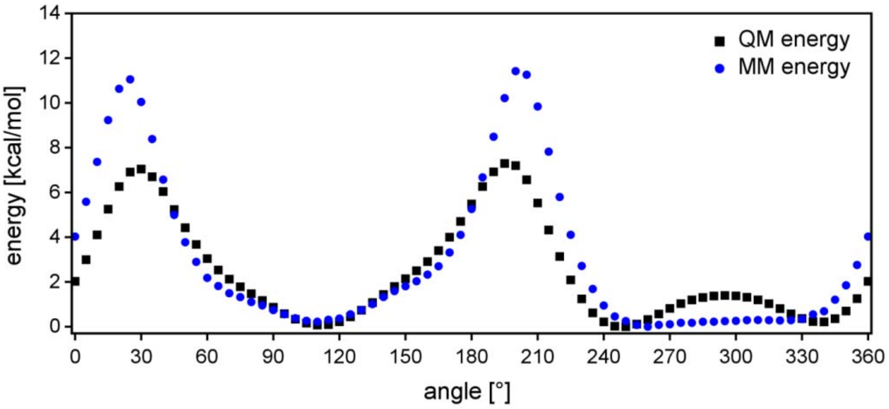

Figure S2. Quantum mechanical (QM) and molecular mechanical (MM) energy profiles for the torsion angle C13-N3-C12-C9. Both energy profiles were normalized to zero.

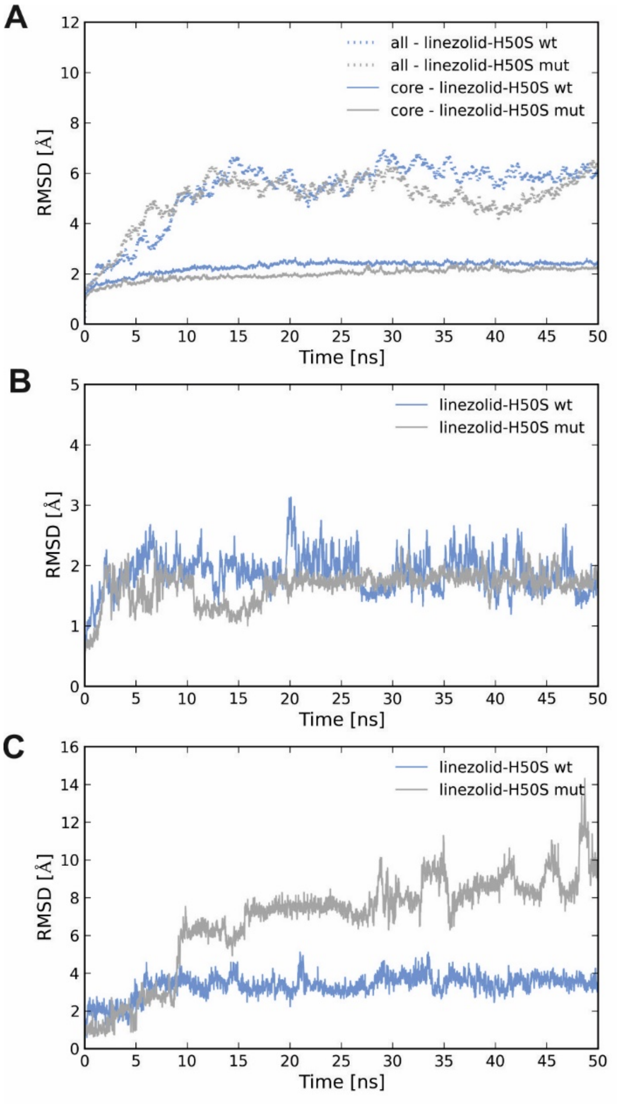

Figure S3: Root mean-square deviations of all-atoms, core-atoms, and the ligand with respect to the starting structure. (A) RMSD of C_α and phosphorous atoms of all residues (dashed lines) and the ‘core residues’ (solid lines) along the MD trajectories of linezolid-H50S$_{wt}$ (blue) and linezolid-H50S$_{mut}$ (grey) complex structures. The ‘core residues’ were defined as those residues with the 90% lowest RMSF of the C_α and phosphorous atoms. (B) RMSD of nucleotides forming the ligand binding site (1st and 2nd shell) along the MD trajectories of linezolid-H50S$_{wt}$ (blue) and linezolid-H50S$_{mut}$ (grey) complex structures. (C) RMSD of linezolid along the MD trajectories of linezolid-H50S$_{wt}$ (blue) and linezolid-H50S$_{mut}$ (grey) complex structures after fitting to the C_α and phosphorous atoms of the ‘core residues’.

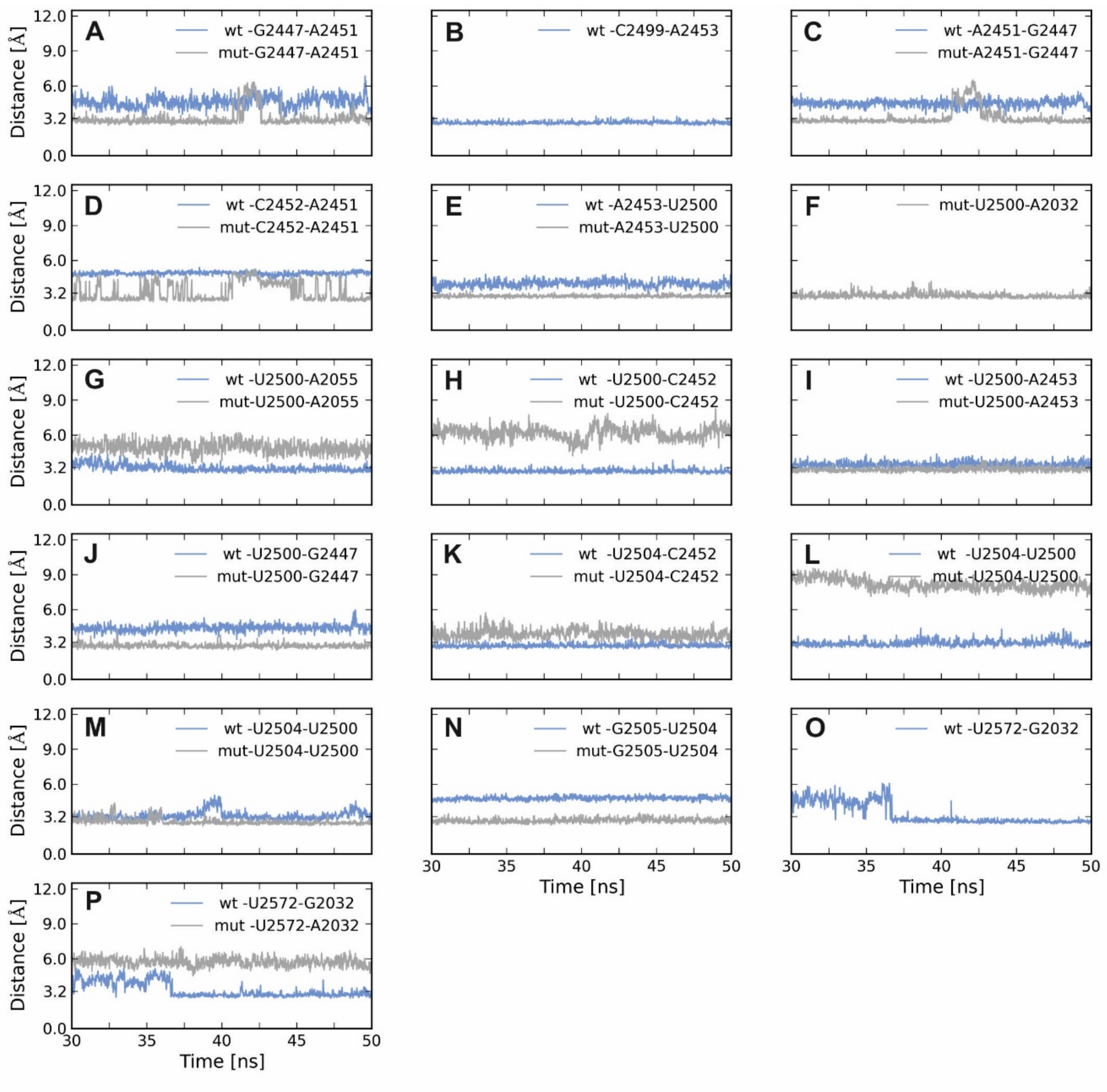

Figure S4: Distances monitoring hydrogen bond formation for linezolid-H50S$_{wt}$ (blue) and linezolid-H50S$_{mut}$ (grey). The monitored distances are ordered according to the number of the acceptor base: i.e. between: (A) G2447@O6 and A2451@N6; (B) C2499@O2 and A2453@N6; (C) A2451@N7 and G2447@N1; (D) C2452@O2P and A2451@O2'; (E) A2453@N1 and U2500@N3; (F) U2500@O2 and A2032@N6; (G) U2500@O2 and A2055@N6; (H) U2500@O4 and C2452@N4; (I) U2500@O4 and A2453@N6; (J) U2500@O5' and G2447@O2'; (K) U2504@O4 and C2452@N4; (L) U2504@O4 and U2500@N3; (M) U2504@O1P and U2500@O2'; (N) G2505@O1P and U2504@O2'; (O) U2572@O2P and G2032@N2; (P) U2572@O4' and G/A2032@N1. In the case of the mutated nucleotides C/A2499 and G/A2032 distances are only shown if the respective hydrogen bond is possible.

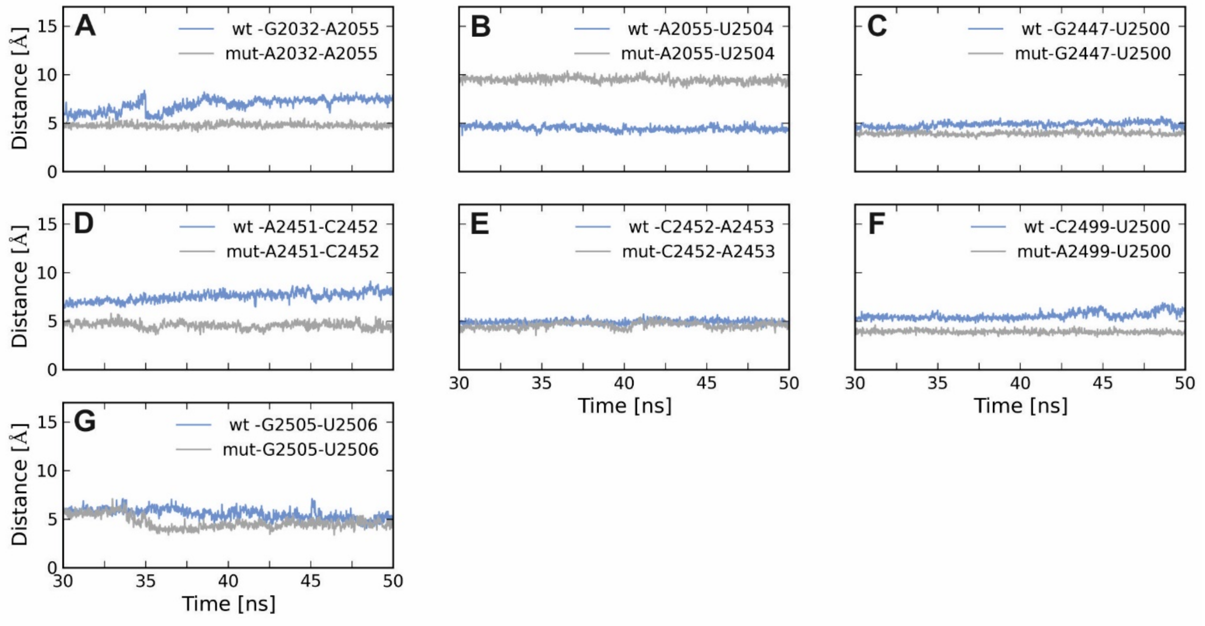

Figure S5: Distances monitoring aromatic stacking interactions for linezolid-H50S$_{wt}$ (blue) and linezolid-H50S$_{mut}$ (grey). The distances were determined between the centers of mass of nucleobases (A) G/A2032 and A2055, (B) A2055 and U2504, (C) G2447 and U2500, (D) A2451 and C2452, (E) C2452 and A2453, (F) C/A2499 and U2500, and (G) G2505 and U2506.

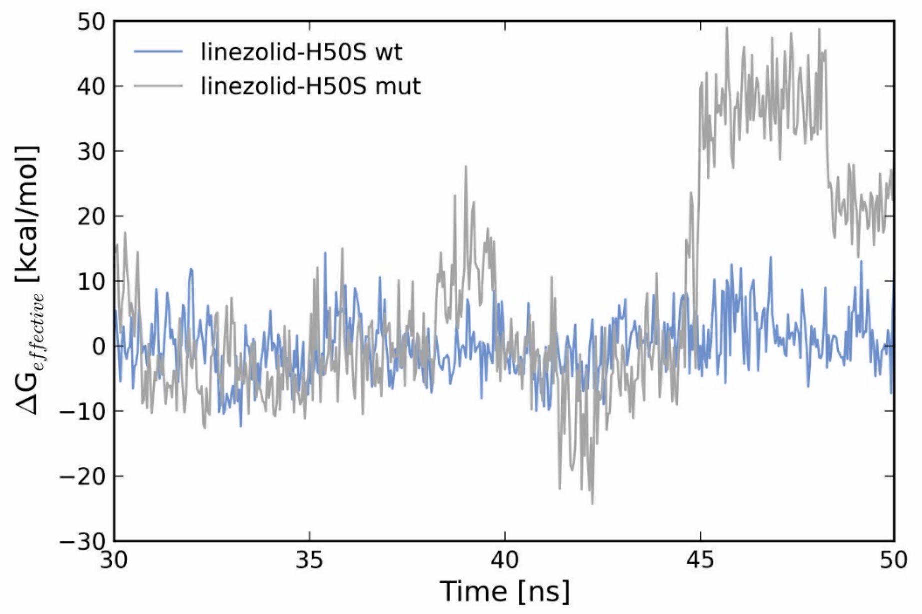

Figure S6: Time series of effective binding energies.

The time series were calculated for 500 snapshots extracted in 20 ps intervals from the last 10 ns of the MD simulations of linezolid-H50S$_{wt}$ (blue) and linezolid-H50S$_{mut}$ (grey). The drifts in the effective binding energies, determined from the slopes of the linear regression lines for linezolid-H50S$_{wt}$ and linezolid-H50S$_{mut}$, are 0.32 kcal mol^{-1} ns^{-1} and 3.62 kcal mol^{-1} ns^{-1}, respectively.

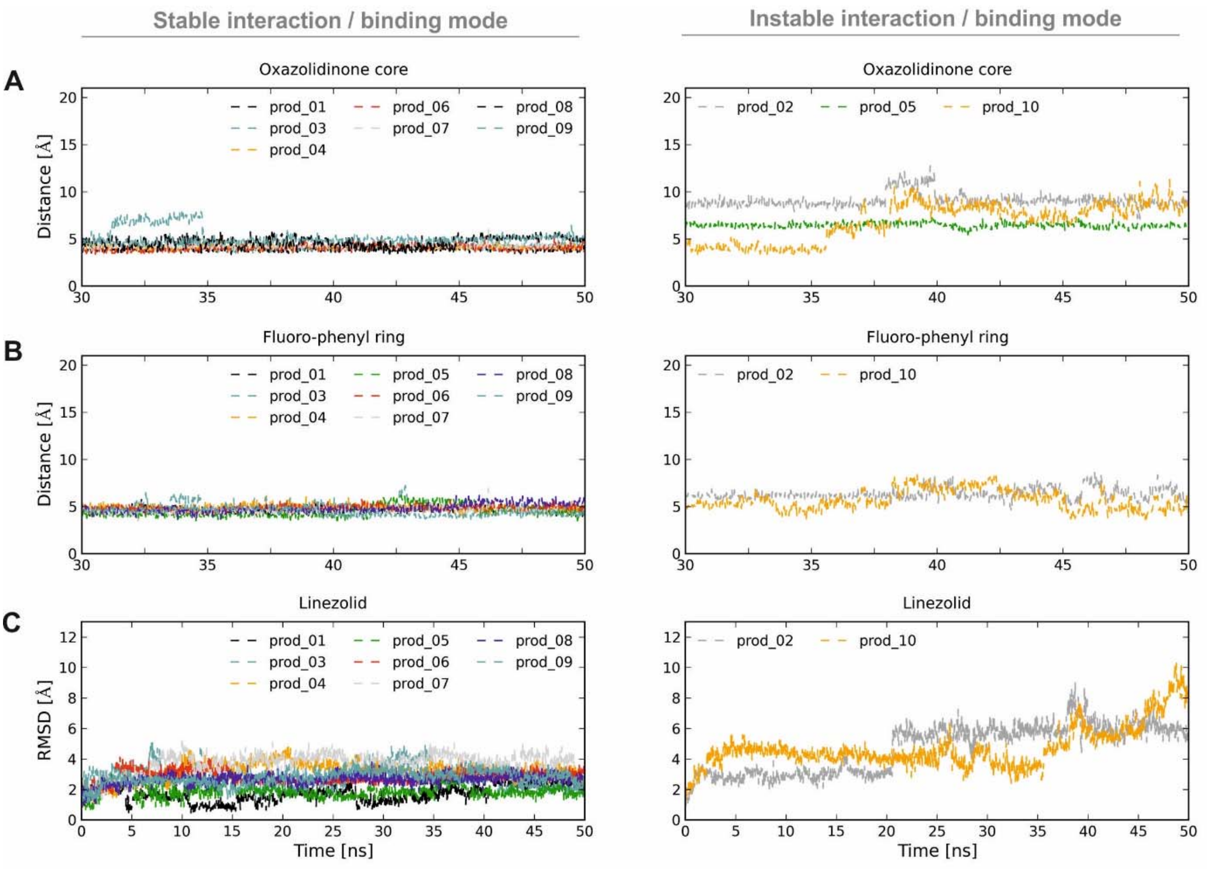

Figure S7: Structural analysis of linezolid-H50S$_{wt}$ control simulations.

(A) and (B) Distances monitoring stacking interactions between the centers of mass of the oxazolidinone core and the nucleobase of U2504 (A) and between the fluoro-phenyl ring and the nucleobase of A2451/C2452 (B). (C) RMSD of linezolid along MD trajectories of linezolid-H50S$_{wt}$ complex structures after fitting to the C$_{\alpha}$ and phosphorous atoms of the 'core residues'. Note that the trajectories are assigned to the left (right) panel if the interaction is stable (instable).

Stacking interactions between the oxazolidinone core or the fluoro-phenyl ring and the nucleobase of U2504 or A2451/C2452, respectively, were defined by a distance cutoff of 5.0 Å from one ring center to another and considered stable if their occupancies attained > 60 % during the last 20 ns of the trajectory. In the case of stacking interactions with the fluoro-phenyl ring, the smallest distance to the nucleobases of A2451 and C2452 was considered, respectively. RMSD values were considered stable if they stayed below 4 Å during the last 10 ns of the respective trajectory.

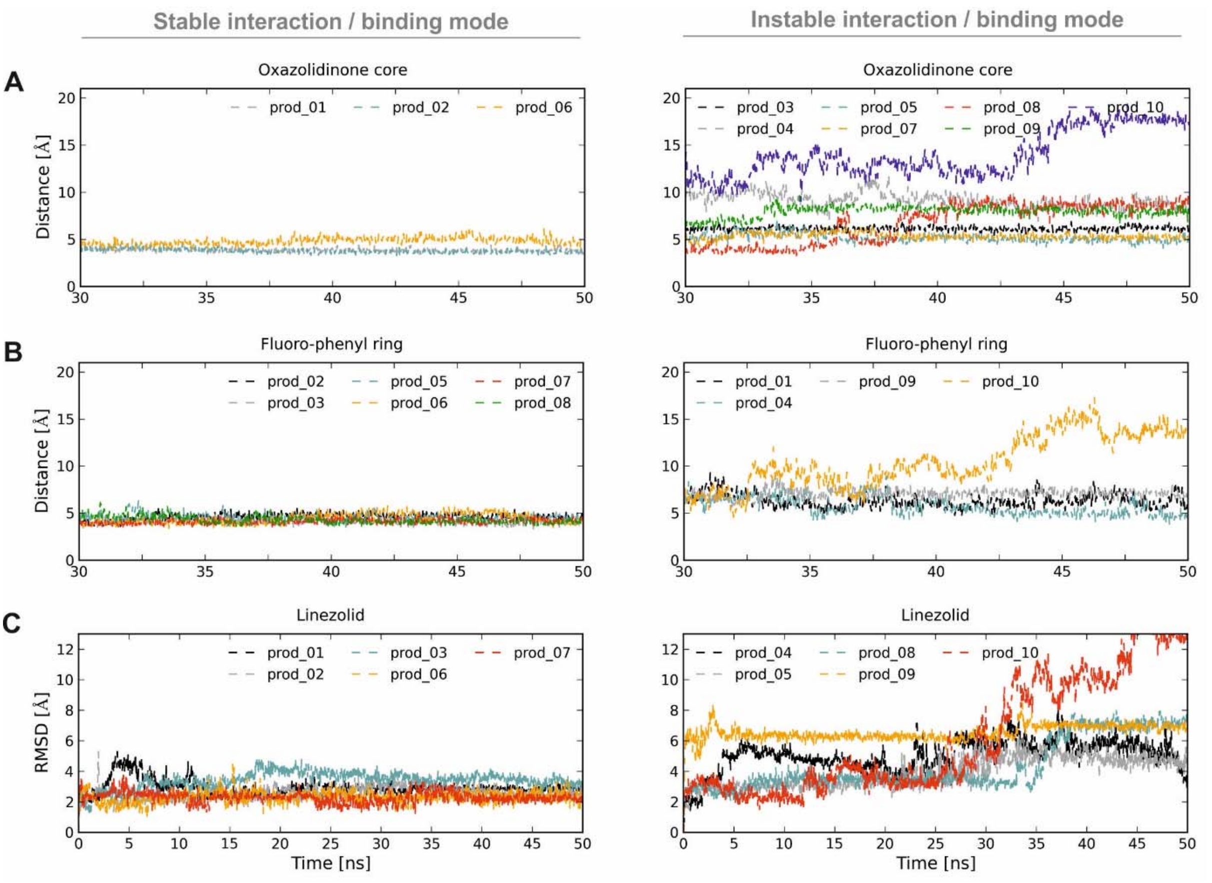

Figure S8: Structural analysis of linezolid-H50S$_{mut}$ control simulations.

(A) and (B) Distances monitoring stacking interactions between the centers of mass of the oxazolidinone core and the nucleobase of U2504 (A) and between the fluoro-phenyl ring and the nucleobase of A2451/C2452 (B). (C) RMSD of linezolid along MD trajectories of linezolid-H50S$_{mut}$ complex structures after fitting to the C_α and phosphorous atoms of the 'core residues'. Note that the trajectories are assigned to the left (right) panel if the interaction is stable (instable).

Stacking interactions between the oxazolidinone core or the fluoro-phenyl ring and the nucleobase of U2504 or A2451/C2452, respectively, were defined by a distance cutoff of 5.0 Å from one ring center to another and considered stable if their occupancies attained > 60 % during the last 20 ns of the trajectory. In the case of stacking interactions with the fluoro-phenyl ring, the smallest distance to the nucleobases of A2451 and C2452 was considered, respectively. RMSD values were considered stable if they stayed below 4 Å during the last 10 ns of the respective trajectory.

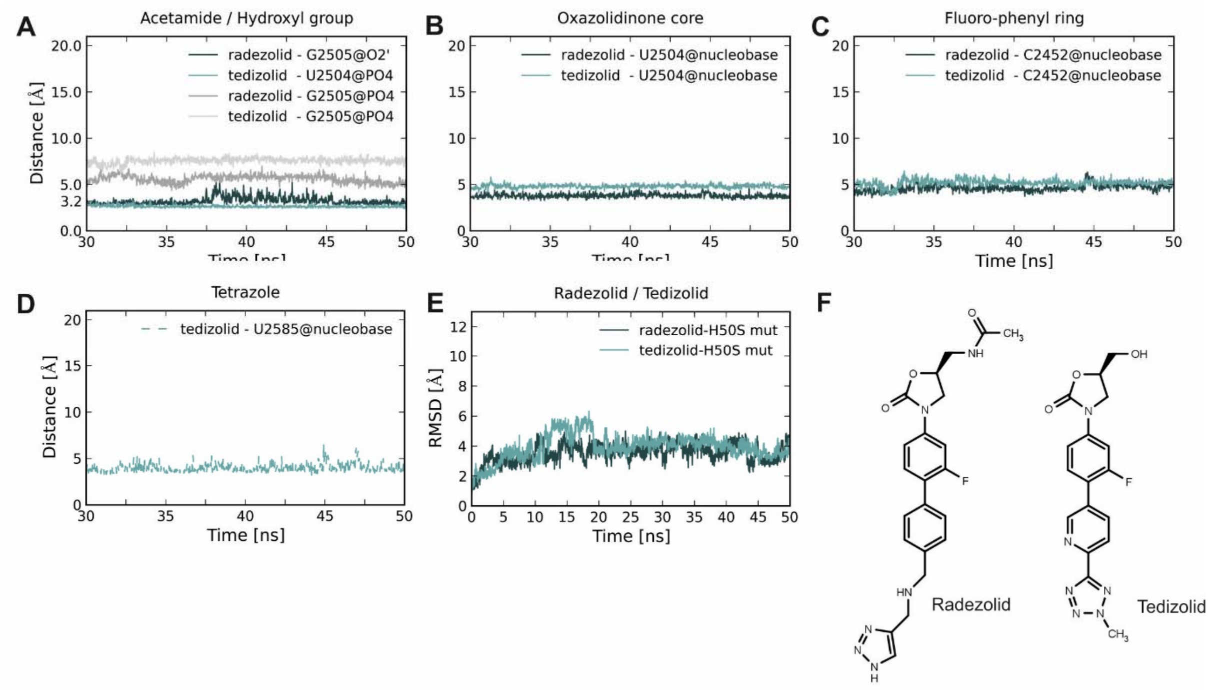

Figure S9: Structural analysis of radezolid-H50S$_{mut}$ and tedizolid-H50S$_{mut}$ simulations. (A) Distances monitoring hydrogen bond formation between radezolid's acetamide NH group and O2' of G2505 (radezolid-H50S$_{mut}$: dark-grey; occupancy 60 %) and between tedizolid's OH group and the oxygens of the phosphate group U2504 (tedizolid-H50S$_{mut}$: turquoise; only the smallest distance found in each snapshot is plotted; occupancy 100 %). Furthermore, corresponding distances with the oxygens of the phosphate group of G2505, as observed in the crystal structure (5), are shown in grey. (B-D) Distances monitoring stacking interactions between the centers of mass of the oxazolidinone core and the nucleobase of U2504 (B), between the fluoro-phenyl ring and the nucleobase of C2452 (C), and between tedizolid's tetrazole ring and U2585 (D). (E) RMSD of radezolid and tedizolid along the MD trajectories of radezolid-H50S$_{mut}$ and tedizolid-H50S$_{mut}$ complex structures after fitting to the C_{α} and phosphorous atoms of the 'core residues'. (F) Chemical structures of radezolid and tedizolid.

Supplementary References

1. Case, D.A., Cheatham, T.E., 3rd, Darden, T., Gohlke, H., Luo, R., Merz, K.M., Jr., Onufriev, A., Simmerling, C., Wang, B. and Woods, R.J. (2005) The Amber biomolecular simulation programs. *J. Comput. Chem.*, **26**, 1668-1688.
2. Frisch, M.J., Trucks, G.W., Schlegel, H.B., Scuseria, G.E., Robb, M.A., Cheeseman, J.R., Scalmani, G., Barone, V., Mennucci, B., Petersson, G.A. *et al.* (2009) Gaussian 09.
3. Davidovich, C., Bashan, A. and Yonath, A. (2008) Structural basis for cross-resistance to ribosomal PTC antibiotics. *Proc Natl Acad Sci*, **105**, 20665-20670.
4. Long, K.S., Munck, C., Andersen, T.M., Schaub, M.A., Hobbie, S.N., Bottger, E.C. and Vester, B. (2010) Mutations in 23S rRNA at the peptidyl transferase center and their relationship to linezolid binding and cross-resistance. *Antimicrob. Agents Chemother.*, **54**, 4705-4713.
5. Ippolito, J.A., Kanyo, Z.F., Wang, D.P., Franceschi, F.J., Moore, P.B., Steitz, T.A. and Duffy, E.M. (2008) Crystal structure of the oxazolidinone antibiotic linezolid bound to the 50S ribosomal subunit. *J. Med. Chem.*, **51**, 3353-3356.
